# Dr. James Lewis Watt

**Published:** 1913-08

**Authors:** 


Dr. James Lewis Watt, L. I. C. Hospital, 1893, a practitioner of Sherman, N. Y., died at the Brooks Memorial Hospital, Dunkirk, June 24, aged 41, of gastric ulcer.

